# Effectiveness of Digital Counseling Environments on Anxiety, Depression, and Adherence to Treatment Among Patients Who Are Chronically Ill: Systematic Review

**DOI:** 10.2196/30077

**Published:** 2022-01-06

**Authors:** Karoliina Paalimäki-Paakki, Mari Virtanen, Anja Henner, Miika T Nieminen, Maria Kääriäinen

**Affiliations:** 1 Research Unit of Nursing Science and Health Management University of Oulu Oulu Finland; 2 Degree Programme of Radiography and Radiation Therapy Oulu University of Applied Sciences Oulu Finland; 3 School of Rehabilitation and Examination Helsinki Metropolia University of Applied Sciences Helsinki Finland; 4 Medical Research Center Oulu Oulu University Hospital and University of Oulu Oulu Finland; 5 Research Unit of Medical Imaging Physics and Technology University of Oulu Oulu Finland; 6 Department of Diagnostic Radiology Oulu University Hospital Oulu Finland; 7 Oulu University Hospital Oulu Finland

**Keywords:** mHealth, mobile health, eHealth, digital health, mobile apps, smartphone apps, web-based, telemedicine, chronic diseases, noncommunicable diseases, web-based interventions, mobile phone

## Abstract

**Background:**

Patients who are chronically ill need novel patient counseling methods to support their self-care at different stages of the disease. At present, knowledge of how effective digital counseling is at managing patients’ anxiety, depression, and adherence to treatment seems to be fragmented, and the development of digital counseling will require a more comprehensive view of this subset of interventions.

**Objective:**

This study aims to identify and synthesize the best available evidence on the effectiveness of digital counseling environments at improving anxiety, depression, and adherence to treatment among patients who are chronically ill.

**Methods:**

Systematic searches of the EBSCO (CINAHL), PubMed, Scopus, and Web of Science databases were conducted in May 2019 and complemented in October 2020. The review considered studies that included adult patients aged ≥18 years with chronic diseases; interventions evaluating digital (mobile, web-based, and ubiquitous) counseling interventions; and anxiety, depression, and adherence to treatment, including clinical indicators related to adherence to treatment, as outcomes. Methodological quality was assessed using the standardized Joanna Briggs Institute critical appraisal tool for randomized controlled trials or quasi-experimental studies. As a meta-analysis could not be conducted because of considerable heterogeneity in the reported outcomes, narrative synthesis was used to synthesize the results.

**Results:**

Of the 2056 records screened, 20 (0.97%) randomized controlled trials, 4 (0.19%) pilot randomized controlled trials, and 2 (0.09%) quasi-experimental studies were included. Among the 26 included studies, 10 (38%) digital, web-based interventions yielded significantly positive effects on anxiety, depression, adherence to treatment, and the clinical indicators related to adherence to treatment, and another 18 (69%) studies reported positive, albeit statistically nonsignificant, changes among patients who were chronically ill. The results indicate that an effective digital counseling environment comprises high-quality educational materials that are enriched with multimedia elements and activities that engage the participant in self-care. Because of the methodological heterogeneity of the included studies, it is impossible to determine which type of digital intervention is the most effective for managing anxiety, depression, and adherence to treatment.

**Conclusions:**

This study provides compelling evidence that digital, web-based counseling environments for patients who are chronically ill are more effective than, or at least comparable to, standard counseling methods; this suggests that digital environments could complement standard counseling.

## Introduction

### Background

Chronic diseases account for 71% of all deaths globally. Furthermore, the recent rapid increase in the number of patients who are chronically ill will heavily burden the health care sector. This review focuses on the use of digital counseling environments among patients with cancer and cardiovascular, musculoskeletal, and colorectal diseases.

Patients who are chronically ill need a variety of counseling approaches at different stages of the disease. Patient counseling, which refers to the interaction between a patient and health care professionals, can strongly support the patient’s sense of responsibility in adhering to their treatment [1]. Most of the novel patient counseling methods, for example, mobile, digital, or ubiquitous counseling, can increase adherence to treatment, which has been worryingly low among patients who are chronically ill [2]. Digital counseling environments can provide peer support through interaction; motivate self-care; and offer understandable, reliable, and up-to-date information to help patients better understand their disease and make lifestyle changes [3-5]. In addition, novel counseling methods can help manage patients’ anxiety and fear, as well as enhance patient safety [2,6,7]. Nevertheless, the current knowledge base regarding digital counseling for anxiety and adherence to treatment among patients who are chronically ill seems to be fragmented, which highlights the need for a comprehensive summary of the available counseling approaches.

Digital, customer-oriented services may improve a patient's quality of life and functionality when the service is accessible regardless of place or time and tailored to the patient’s specific needs [8,9]. Various technologies now enable the provision of such services, which can provide individual counseling to patients at the correct time and in an appropriate manner [10-12]. The provision of materials in different formats promotes tailored counseling approaches [11-15], with previous research demonstrating that patients value inclusivity, comprehensibility, availability, and flexibility in these services [4,13,16-18].

In recent years, digitalization has offered numerous opportunities for providing health care through digital channels. The World Health Organization defines digital health as “a broad umbrella term encompassing e-health, as well as developing areas such as the use of advanced computer sciences.” Mobile health (mHealth) is a subarea of digital health, and is described as “the use of wireless mobile technologies for health,” whereas another subarea, ubiquitous health, is defined as services delivered through ubiquitous technologies such as tags, sensors, and biometric devices [19]. The main objective of digital health could be described as using various technologies to support the achievement of health goals through the internet. However, the realm of digital health is wide and, as such, various terms have been applied in digital health research. This review focuses on web-based solutions and mobile apps that integrate knowledge sharing to create participative elements for the patient. Studies focusing on SMS text messaging and gaming were excluded.

According to the World Health Organization, digital and mobile technologies support health care systems through targeted and untargeted patient communication, patient-to-patient communication, personal health tracking, and citizen-based reporting. An important objective of digital health interventions is the widespread promotion of positive changes in behavior to prevent the onset of chronic disease.

The impacts of various digital health interventions on the management of chronic diseases, especially diabetes mellitus [20-26], cardiovascular diseases, and cancer, have been studied extensively by systematic reviews during recent years [10,27-47]. However, many of the studies that have been reviewed suffer from methodological shortcomings, that is, insufficient power to detect changes in outcomes and relatively short study duration [31]. This has led to a fragmented knowledge base, and a comprehensive description of the available digital health solutions—along with their effectiveness—is needed to further develop counseling for patients who are chronically ill. Research implications for future eHealth studies have recently been identified, categorized, and prioritized [48]. For example, randomized controlled trial (RCT) studies with large sample sizes and long follow-up periods, along with investigations of the cost-effectiveness and user acceptance of eHealth interventions, should be conducted in the near future. Furthermore, decision-makers will need an improved understanding of which components of the studied interventions, for example, frequency, duration and delivery mode, or patient characteristics, contribute most to the overall eﬀectiveness of an eHealth intervention. Ethical aspects, intervention safety, and translation of ﬁndings into a practical context were also identified as necessary research elements [48].

### Objectives

This systematic review aims to identify and synthesize the best available evidence on the effectiveness of digital counseling environments for managing anxiety, depression, and adherence to treatment among patients who are chronically ill.

This review answers the following question: What is the effectiveness of the digital counseling environments compared with control (eg, usual care) on anxiety and depression and clinical outcomes related to adherence to treatment?

## Methods

### Systematic Review

A systematic review of RCTs was conducted according to the Centre for Reviews and Dissemination and Joanna Briggs Institute guidelines [49,50]. The research adhered to the PRISMA (Preferred Reporting Items for Systematic Reviews and Meta-Analyses) statement [51] regarding the reporting of evidence.

### Inclusion and Exclusion Criteria

The selection of studies was based on predefined inclusion and exclusion criteria, which are reported in the patient, intervention, comparison, and outcome format [50]. The review considered studies that included participants who were adult patients aged ≥18 years with chronic diseases; described interventions that were digital (mobile, web-based, or ubiquitous) counseling approaches; and reported outcomes that were patient outcomes (primary outcomes), that is, anxiety, depression, or adherence to treatment, and clinical indicators (secondary outcomes) related to adherence to treatment. The comparator was no treatment, standard care, or another type of intervention. All RCTs and quasi-experimental studies published in English, Finnish, or Swedish from 2008 to 2020 were considered; this specific time period was selected to reflect the growth and adoption of digital technologies.

Studies focusing on patients aged <18 years or describing patients with psychiatric disorders or substance abuse problems were excluded. Furthermore, studies focusing on traditional counseling, SMS text message counseling, or eHealth game development were excluded. Studies were also excluded if they measured any outcomes other than those defined in the inclusion criteria or were protocols, reviews, editorial papers, discussions, recommendations, or parts of books.

### Search Strategy

Systematic literature searches were conducted across 4 electronic databases (CINAHL, PubMed, Scopus, and Web of Science) in May 2019, after which the search was complemented for the years 2019-2020 in October 2020. The reference lists of the included studies were screened for studies that may be relevant to the study objective, yet were not identified during the systematic literature search. An information specialist assisted the researchers in forming a search strategy and conducting the literature search. The search strategy for different databases is presented in Multimedia Appendix 1.

### Study Selection

**Figure 1:**
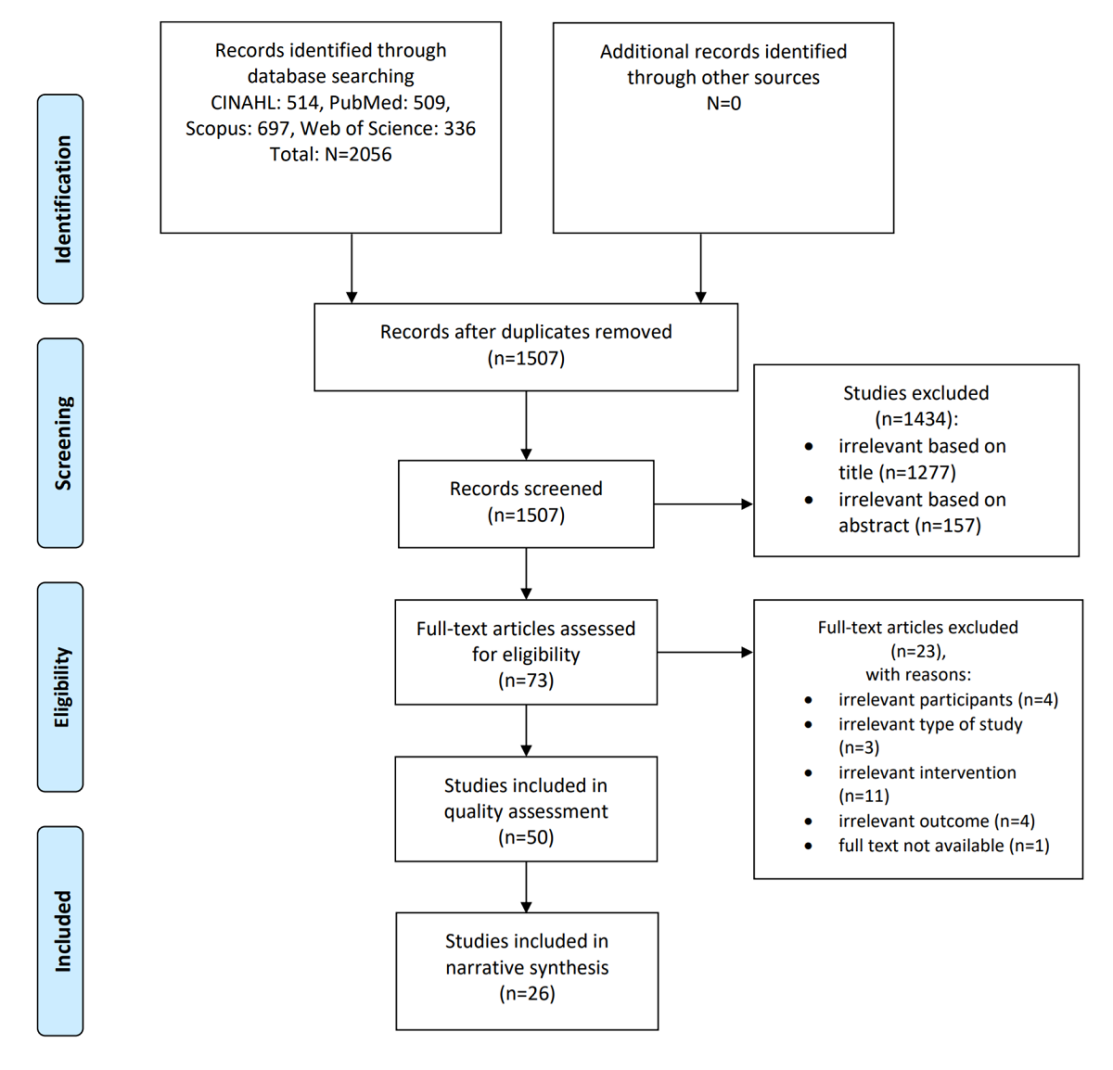
PRISMA (Preferred Reporting Items for Systematic Reviews and Meta-Analyses) 2009 flow diagram of study selection process. CINAHL: Cumulative Index to Nursing and Allied Health Literature.

A total of 2056 publications were retrieved during the database searches. These publications were then imported into Zotero reference manager software (Corporation for Digital Scholarship). From the 2056 publications, 549 (26.7%) duplicates were removed. Of the 1507 studies remaining, 1434 (95.16%) were excluded after title and abstract screening by 2 independent researchers (KPP and MK) using predefined inclusion criteria, leaving 73 (4.84%) full-text articles relevant to the study objectives. Minor disagreements between the reviewers were resolved, and the researchers reached agreement. At the completion of the screening process, of the 73 studies, 50 (68%) met the inclusion criteria and were included in the critical appraisal. A PRISMA flow diagram was used to present the study selection process (Figure 1).

### Critical Appraisal

The methodological quality of the 50 selected studies was independently assessed by 2 researchers (KPP and MK) using a standardized Joanna Briggs Institute critical appraisal tool for RCTs and quasi-experimental studies [52]. The methodological quality was evaluated by assigning points to each criterion of the appraisal tool. Studies that scored at least 60% (8/13 points for RCTs and 5/9 points for quasi-experimental studies) across the appraisal criteria were included in the review. Of the 50 selected studies, 26 (52%) were included in the final review, whereas 24 (48%) were excluded based on poor blinding, unreliable measurement of outcomes, or inappropriate statistical analysis. Critical appraisal of the selected randomized controlled trial studies is presented in Multimedia Appendix 2 [53-76].

In the critical appraisal of the selected quasi-experimental studies (2/26, 8%), both scored 8 points out of 9. Each study had a control group, and there were no differences among the participants in the compared groups. Other than the intervention of interest, there were no differences between the groups in terms of care received. Multiple measurements of the outcomes both before and after the intervention were collected in the same way in both studies, and appropriate statistical analysis was conducted. The only unclear criterion concerned whether the follow-up was complete, and if not, whether differences between the groups in terms of their follow-up were adequately described and analyzed.

### Data Extraction

Data from the original studies included in the review were extracted to meet the Centre for Reviews and Dissemination information requirements [49]. The first author (KPP) entered the extracted data into a standardized form (Multimedia Appendix 3 [53-78]) that also included the quality assessment scores. The second author (MV) confirmed the extracted data.

Because of the heterogeneity of outcomes reported in the identified RCTs, a meta-analysis was not possible [49]. Narrative synthesis was used to answer the research question.

## Results

### Characteristics of the Included Studies

A total of 26 studies were included in this review: 20 (77%) RCTs [53-72], 4 (15%) pilot RCTs [73-76], and 2 (8%) quasi-experimental studies [77,78] published from 2010 to 2020 in English in 13 countries. Details of the included studies are described in Multimedia Appendix 3.

### Participants

The participants in the included studies were adult patients aged ≥18 years with a range of diseases: various cancers [53,63,65,66,69,70,73,75-77], along with cardiovascular [54,56-58,64,68,71,72,74], musculoskeletal [59,62], colorectal [55,60,61], and kidney diseases [78]. The sample size ranged from 29 to 1000 patients, with, of the 26 studies, 9 (35%) having enrolled fewer than 99 participants [58-60,62,64,68,73,76,78], 9 (35%) having enrolled 100-200 participants [53,54,61,66,69,70,74,75,77], 7 (27%) having enrolled 201-500 participants [55-57,63,65,67,72], and 1 (4%) having enrolled 1000 participants [71]. The follow-up period ranged from days to 12 months, more specifically, 3 months or less in 46% (12/26) of the studies [59-61,64,66,68-70,73,74,76,78], 3-6 months in 23% (6/12) of the studies [58,62,63,65,67,71], and 6-12 months in 31% (8/26) of the studies [53-57,72,75,77].

### Interventions

#### Overview

The patient counseling environments described in the original publications included websites [53,55-57,59,62,63, 65,66,72,75,76], mobile apps [54,60,61,67-71,73,74,77,78], or a combination of both [58,64]. Websites could be accessed with all devices, whereas the mobile apps were accessible with either a mobile phone or a tablet. The mobile apps were designed to be both iOS and Android compatible [54,61,64,74], only iOS compatible [58,60,67,73], or only Android compatible [68,77,78]. The interventions described in this review were heterogeneous, with detailed information provided in Multimedia Appendix 3. The interventions are described according to the Template for Intervention Description and Replication checklist [79].

#### Websites

Of the 26 studies, websites were the primary counseling approach in 12 (46%). Of these 12 studies, 6 (50%) focused on patients with cancer [53,63,65,66,75,76], 1 (8%) focused on patients with colorectal disease [55], 2 (17%) concerned patients with musculoskeletal disease [59,62], and 3 (25%) covered patients with cardiovascular disease [56,57,72]. The format, amount, and use of the counseling materials in the presented websites varied among the studies.

Counseling materials provided disease- or condition-specific information in different formats. The materials were gathered as learning material libraries [55,65,66,72,75,76], link collections [53,63,72,75], and patient stories [56,72,75].

In addition, 92% (11/12) of the presented websites included information-processing functionalities [53,55-57,59,62,63, 65,66,72,76]. Participants were encouraged to assess, self-monitor, and report personal health data such as heart rate, blood pressure, blood glucose level, symptoms, distress, medication adherence, daily exercise, and diet [53,55,57,59,63,65,66,72,76] and fill in web-based medical, risk factor, and lifestyle forms [55-57,65,66]. Adherence to treatment was followed by learning tasks [56,66], an e-notebook or diary [53,55,62,63,66], and action plans [57,72,76]. Participants also received personalized advice, recommendations, and feedback based on their activity and self-reports [53,55-57,59,63,65,66]. All the websites described in 100% (12/12) of the studies included personalized content [53,55-57,59,62,63,65,66,72,75,76], that is, counseling, recommendations, and feedback based on the participants’ inputs and responses.

Web-based patient–provider counseling was integrated into 58% (7/12) of the presented websites: web-based communication occurred through e-messages [53,55,57,65,66,75] and videoconferencing [75]. For 8% (1/12) of the websites, participants had the option to save an updated list of questions for the health care team [76]. Furthermore, 25% (3/12) of the studies included a component, that is, anonymous web-based forum group discussions [53,62,76] and blog [53], through which users could share their experiences with other patients.

Website use and activity were measured in 17% (2/12) of the included studies [53,63]; more specifically, these studies applied the following website analytics: total hits per user session [53], hits on program modules and pages [53], total viewing time [63], number of website log-ins per person [53,63], number of measures uploaded, amount of e-messages sent, and number of diary notes and posts in blog [53].

#### Mobile Apps

Of the 26 studies, mobile apps were the primary approach used in 12 (46%). Of these 12 studies, 5 (42%) focused on patients with cardiovascular diseases [54,67,68,71,74], 4 (33%) covered patients with cancer [69,70,73,77], 2 (17%) focused on patients with colorectal diseases [60,61], whereas 1 (8%) was conducted on patients requiring hemodialysis [78].

The format, amount, and use of the counseling materials in the mobile app varied among the studies. In addition to counseling materials, 67% (8/12) of the presented apps included information-processing functionalities [54,60,61, 67,68,70,71,74]. Participants were encouraged to self-monitor and report health data such as blood pressure, physical activity, diet, medication adherence, symptoms, and sleep [54,60,67,68,70,71,78]. Participants would then receive personalized recommendations, feedback [54,60,67,68,70,71,74,78], and timed notifications [61,67,68,71,77,78] based on their activity and the information they entered into the app. Furthermore, of the 12 apps, 1 (8%) included a personal health record [74] that a patient could update with laboratory test results and use as a risk assessment tool. The input data were also used as a clinical decision support tool by doctors [74]. The presented apps promoted adherence to treatment through daily or weekly challenges [54,60,67], a diary feature [54,78], and homework exercises [69]. Of the apps, 100% (12/12) provided personalized content [54,60,61,67-71,73,74,77,78], that is, counseling, recommendations, feedback, or notifications based on the participants’ inputs and responses.

Of the 12 apps, web-based counseling was integrated into 3 (25%). Of these 3 apps, 1 (33%) included 60 minutes of individual counseling with a registered dietitian [54], 1 (33%) involved conversational messages that were responded to by artificial intelligence [67], and 1 (33%) included counseling through a bulletin board and SMS text messaging with the researcher [78].

App use was measured and reported in 33% (4/12) of the studies. For example, the patient satisfaction rate was calculated [61,71,74]; the frequency of app use was measured [70,71]; and the usability, feasibility, and acceptability of the app were investigated [74]. Of the 4 studies, 2 (50%) reported that the apps were rated as user friendly and easy to use, as well as helpful or indispensable [61,74], whereas only 15% of the participants in 1 (25%) study perceived the app to be very useful, with more than half of the participants perceiving the app to be of little use [71].

#### Combination of Website and Mobile App

Of the 26 studies, 2 (8%) concerning patients with cardiovascular disease used the combination of a website and mobile app as the primary approach for improving patients’ mental health and adherence to treatment [58,64]. The format, amount, and use of the counseling materials varied between these 2 reports.

In addition to counseling materials, both studies reported that the presented intervention included information-processing functionalities. For example, participants used their mobile phone to enter health data such as blood pressure, blood glucose level, medication adherence, and diet [58,64]. Adherence to treatment was promoted by learning tasks and homework [64], action plans for lifestyle change [64], and reminders for self-monitoring [58]. Participants received automated, personalized recommendations and feedback based on their activity and input [64]. Furthermore, of the 2 interventions, 1 (50%) enabled the sharing of data, that is, a patient could share their personal health record with family members, caregivers, and health professionals [58]. Both the described interventions included personalized content [58,64].

### Outcomes

#### Overview

Of the 26 studies included in this review, 13 (50%) measured anxiety and depression [53,55,56,62,63,65,66,69, 70,73,74,76,77], 9 (35%) measured adherence to treatment [54,55,57,59-61,71,75,78], and 9 (35%) studied how the digital environment affected ≥1 clinical outcomes related to adherence to treatment [54,57,58,64,67,68,71,72,78]. The scales used to measure these outcomes are described in Textbox 1.

**The scales used to measure the outcomes.**

**Outcomes and scales**

- Anxiety and depression
  - Hospital Anxiety and Depression Scale [53,55,62,63,65,66,69,70,73,76,77]
  - General Anxiety Disorder Scale-7 [56]
  - Hamilton Anxiety Rating Scale [69]
  - Distress Thermometer [66,76]
  - Impact of Events Scale [76]
  - EuroQol [74]
  - EuroQol 5-Dimensional Questionnaire, Youth Version [74]
  - Patient Health Questionnaire-9 [56,69]
- Adherence to treatment
  - Mediterranean Diet Score [54]
  - Compliance Questionnaire [55]
  - Medical record reviews [75]
  - Framingham Risk Score [57]
  - Numerical scale from 0 to 10 [59]
  - Mean adherence to predefined bundle of patient-dependent elements [60]
  - Compliance with the first low-fiber dietary change and duration of use of the clear liquid diet and bowel cleanliness using 3 scales: the modified Aronchick scale, the Ottawa Bowel Preparation Scale, and the Chicago Bowel Preparation Scale [61]
  - Morisky Medication Adherence Scale [71]
  - Compliance of Patient Role Behavior Tool [78]
- Clinical indicators related to adherence to treatment
  - Blood pressure [54,57,58,64,67,71,72]
  - Weight [54,57,64,78]
  - Total cholesterol [54,57,64,68]
  - High-density lipoprotein cholesterol [54,57,64,68]
  - Low-density lipoprotein cholesterol [54,57,64,68]
  - Triglycerides [54,64,68]
  - Glycosylated hemoglobin level [54,57]
  - High-sensitivity C-reactive protein [54,57]
  - Serum glucose values [64]
  - Frequency of alcohol consumption and frequency of smoking [57,58,64,71]
  - Frequency of exercise [57,58,64]
  - Exercise stress test [64]
  - Alanine aminotransferase, creatinine, and plasma carotenoids [57]

#### Anxiety and Depression

An analysis of the 26 identified studies revealed that 3 (12%) reported a statistically significant reduction in anxiety and depression. A few of the presented websites significantly reduced anxiety and depression among patients with cancer [53,65], and a mobile app decreased anxiety and depression among patients with cardiovascular disease [74] in comparison with the control groups.

In an RCT of web-based self-management support for 167 patients with breast cancer, the web choice group reported significantly lower anxiety (mean difference –0.79, 95% CI –1.49 to –0.09; *P*=.03) and depression (mean difference –0.79, 95% CI 1.18 to –0.05; *P*=.03) scores than the usual care group [53]. A web-based tailored program for 273 cancer survivors was able to significantly decrease patients’ Hospital Anxiety and Depression Scale (HADS) score (mean difference –0.90, intervention group SD 3.83-2.79 vs control group SD 3.86-2.59; 95% CI –1.51 to –0.29) compared with the control treatment [65]. In a pilot RCT that included 209 patients with atrial fibrillation [74], a mobile app reduced patients’ anxiety and depression (*P*=.02) compared with the group that did not use the app.

Of the 26 studies, in 9 (35%), the experimental group exhibited positive changes in anxiety and depression; however, these changes were not statistically significant when compared with the results of the control group [55,56,62,63,66,69,70,76,77]. Of the 24 RCTs, 2 (8%) [63,66] assessed the effectiveness of an informational website in reducing distress among patients with cancer. Among 337 patients with breast cancer [63] and 129 patients newly diagnosed with cancer [66], the mean levels of anxiety or depression did not significantly differ between the intervention and control groups. However, the entire study population exhibited a significant decrease in the HADS score in 50% (1/2) of these studies (*P*=.03) [66]. According to the visual analog scale score (which ranges from 0 to 10), the intervention group showed significantly lower levels of distress than the control group (mean difference –0.85, 95% CI –1.60 to –0.10; *P*=.03) 2 months after an intervention [66].

In a pilot RCT [76] for patients with advanced ovarian cancer, no differences between the intervention and control groups were observed for any distress measure, although the group using the patient-centered, information-based website demonstrated lower, albeit nonsignificant, general distress as measured by the Distress Thermometer. In a double-center study of patients with mild to moderate ulcerative colitis [55], the patients in the control group in Denmark showed a significant improvement in depression (*P*=.01) compared with those in the intervention group, whereas the patients in Ireland who had used the tested website demonstrated a significant improvement in anxiety (*P*=.02) compared with those in the control group [55]. Of the 24 RCTs, 2 (8%) studies, with 1 (50%) that included patients with implantable cardioverter defibrillators [56] and 1 (50%) that included patients undergoing lumbar spine fusion [62], found that a web-based platform for anxiety did not significantly affect patients’ anxiety and depression. Furthermore, RCTs investigating the effect of mobile apps on anxiety in patients with incurable cancer [69] and patients undergoing breast cancer chemotherapy [70] reported that both study groups experienced improvements in anxiety and depression, but no significant between-group differences existed. However, subsequent analyses of a subgroup of patients with severe baseline anxiety revealed that patients using the tested app showed greater improvements in the Hamilton Anxiety Rating Scale score (mean difference 7.44, SE 3.35; *P*=.04) and the HADS score (mean difference 4.44, SE 1.60; *P*=.01) than those in the control group [69]. A Taiwanese quasi-experimental study reported that a web-based intervention did not significantly improve distress, anxiety, and depression among breast cancer survivors [77]. A pilot RCT study of female patients undergoing surgery for breast cancer [73] reported similar between-group anxiety and depression scores both preoperatively and immediately after surgery; however, the control group, which did not have access to the additional information provided by the mobile app, showed significantly lower anxiety and depression scores (*P*=.03 and *P*=.02, respectively) 7 days after surgery.

#### Adherence to Treatment

Of the 26 studies, 4 (15%)—2 (50%) of which tested a website [55,59] and 2 (50%) of which presented a mobile app [74,78]—reported statistically significant improvements in adherence to treatment in the intervention group compared with the control group. Lambert et al [59] evaluated the effect of a home exercise website with remote support on self-reported exercise adherence among 80 people with upper or lower limb musculoskeletal conditions. The mean between-group difference for self-reported exercise adherence was 1.3 (11 points; 95% CI 0.2-2.3) in favor of the intervention group, which was a statistically significant result (*P*=.01). A double-center RCT in Denmark and Ireland reported better ulcerative colitis compliance among Danish patients who had used the tested websites than among those in the control group after 12 months, with adherence to 4 weeks of acute treatment also significantly better among the patients in the intervention group (73% compared with 42% among patients in the control group; *P*=.005) [55]. At the Irish center, the patients in the intervention group also showed better adherence to 4 weeks of acute treatment than those in the control group (73% vs 29%; *P*=.03). Moreover, a mobile app for patients with atrial fibrillation significantly improved drug adherence (*P*<.001) and anticoagulant satisfaction (*P*=.01) compared with usual care [74]. In a quasi-experimental study of self-management among 84 patients requiring hemodialysis, the use of a mobile app significantly improved self-efficacy compared with the results from patients in the control group (mean 4.79, SD 3.51 vs mean −1.05, SD 2.05; *t*_82_=−9.30; *P*<.001). Treatment compliance also significantly increased in the experimental group (mean 11.57, SD 7.63) compared with the control group (mean −1.74, SD 2.71; *t*_82_=−10.66; *P*=.001) [78].

Of the 26 studies included in this review, 6 (23%) found no significant between-group differences in adherence to treatment, and 2 (8%) evaluated how the use of a mobile app affects adherence to care among patients with cardiovascular disease [54,71]. The presented asynchronous dietary counseling mobile app resulted in a significantly larger proportion of participants who complied with the Mediterranean diet (Mediterranean Diet Scale score ≥9) over time (*P*<.001); however, no significant between-group differences were discerned. An RCT focusing on a mobile app for patients who had undergone surgical coronary revascularization did not reveal any significant between-group differences in mean medication adherence scores (mean difference 0.052, 95% CI –0.087 to 0.191; *P*=.46) at the 6-month follow-up point [71]. Keyserling et al [57] investigated whether a web-based lifestyle (n=193) and a medication intervention (n=192) can reduce coronary heart disease risk. Both intervention formats reduced coronary heart disease risk through the 12-month follow-up period; however, no significant between-group differences were found [57].

Helzlsouer et al [75] reported that a web-based navigation program for newly diagnosed low-income patients with breast cancer did not significantly affect treatment completion compared with the control group. No significant between-group differences in the assessed measures of adherence were observed in 20% (2/10) of the RCTs of mobile apps, the first of which aimed to improve adherence as part of a recovery program after colorectal surgery [60] and the second aiming to improve adherence to bowel cleanliness among patients who had undergone colonoscopy [61]. However, both the apps were rated as user friendly and a better alternative to paper instructions (*P*<.001). [61]

#### Clinical Outcomes Related to Adherence

Of the 26 studies, 3 (12%) concerning patients with cardiovascular disease—1 (33%) presented the combination of a smartphone app and a website [58], 1 (33%) studied the effectiveness of a website [72], and 1 (33%) presented a mobile app [68]—reported that the intervention group differed significantly from the control group in terms of clinical indicators related to adherence to treatment.

In a 6-month–long RCT that included 95 participants with hypertension, the combination of a smartphone app and website yielded significant improvements in clinical indicators related to adherence among patients in the intervention group compared with those in the control group [58]. More specifically, the results showed reduced consumption of cigarettes (*P*<.001) and decreased systolic and diastolic blood pressure levels (baseline: 140.6/89.4 mm Hg; end of study: 136.5/83.9 mm Hg) in the patients in the intervention group compared with those in the control group. Furthermore, the frequency at which the patients in the intervention group achieved blood pressure control increased from 45% to 59%. Similarly, e-counseling for patients with hypertension (n=264) resulted in a significant reduction in systolic blood pressure after 12 months in the patients in the intervention group compared with those in the control group (–10.1, 95% CI –12.5 to –7.6 mm Hg vs –6.0, 95% CI –8.5 to –3.5 mm Hg; *P*=.02) [72]. A 12-week smartphone app intervention for 57 patients with cardiovascular disease led to significant reductions in both triglyceride and total cholesterol levels in the intervention group compared with the control group (*P*=.02 and *P*=.01, respectively) [68]. In the same study, medication adherence also significantly increased in the intervention group (43.33% vs 82.14%; *P*=.002), whereas the control group only showed a minor increase (30% vs 37.93%; *P*=.52). This between-group difference was statistically significant (82.14% vs 37.93%; *P*=.001). No significant between-group changes were found with respect to low-density lipoprotein and high-density lipoprotein levels [68].

Digital health interventions for 80 patients with acute coronary syndrome [64], 100 patients with cardiovascular disease [54], and 84 patients requiring hemodialysis revealed improved weight loss in the intervention group compared with the control group (mean −5.1, SD 6.5 kg vs mean −0.8, SD 3.8 kg; *P*=.02 [64]; 1.5 kg vs 1.4 kg; *P*=.04 [54]; and mean −0.56, SD 0.88 vs mean 0.05, SD 1.08; *P*=.005 [78], respectively). Among the patients with cardiovascular disease, the digital health intervention did not significantly affect systolic blood pressure [54,57,64,67,71], diastolic blood pressure [54,64,71], lipids [54,57,64], blood glucose level [64], glycosylated hemoglobin level, C-reactive protein [54], or smoking frequency [57,71].

## Discussion

### Principal Findings

This systematic review identified and synthesized the best available evidence regarding how effective digital counseling interventions are at managing anxiety, depression, and adherence to treatment among patients who are chronically ill.

The results indicate that an effective digital counseling environment includes both high-quality educational material, possibly enriched with multimedia elements, and activities that engage participants. Because of the heterogeneity of the studies included in this review, it was impossible to determine which type of digital intervention was the most effective at managing anxiety, depression, and adherence to treatment. Furthermore, determining the aspects responsible for changes in self-management was difficult. Overall, digital, web-based counseling environments designed for patients who are chronically ill seem to be more effective than, or at least comparable to, standard counseling methods. This indicates that well-accepted digital environments could complement standard counseling. Patients should be afforded a variety of web-based educational resources that correspond to their care objectives and needs. These services should also be provided at an appropriate time to ensure maximum benefits [39]. Previous reviews have identified the highly participative features of mHealth interventions, for example, reminders and continuous feedback, patient-centeredness, individually tailored content, and patient–provider communication, to be a large advantage of these services [28,31,34,41,80,81]. Furthermore, it has previously been suggested that digital environments have the potential to increase patient involvement, empowerment, and security through increased knowledge, symptom management, participation, engagement, and improved clinician–patient communication [82,83]. These types of services also do not depend on location, which will improve access to care for patients in remote locations where other services may not be available and, therefore, counteract care inequity. In light of the COVID-19 pandemic, digital environments can also support patients who are chronically ill and living in isolated circumstances [9].

Digital counseling environments can enhance clinical practice and care by empowering patients with chronic disease self-management, reducing dependency on health care professionals, and possibly changing the chronic disease course in the long term. Furthermore, digital counseling environments can be accommodated and used for other patient groups by enhancing diagnostic examination success and optimizing care procedures.

Nevertheless, digital environments can also contribute to care inequity if certain patients do not have the ability or resources to access digital environments. Moreover, digital environments can cause ambivalence and uncertainty if patients lack the digital skills and knowledge of how to use these environments [82].

Surprisingly, all the studies included in this review were based on basic technologies, that is, internet-based environments, websites, and mobile apps. There were no reports of interventions that applied emerging technologies such as augmented reality, virtual reality, mixed reality, or 360° virtual reality solutions. Furthermore, none of the presented digital counseling approaches used ubiquitous elements, for example, tags or sensors.

### Effects of Digital Counseling Environment on Patient and Clinical Outcomes

Digital interventions significantly improved anxiety and depression among patients with cancer [53,65] and cardiovascular disease [74]. Positive, albeit statistically nonsignificant, changes in anxiety and depression were also measured among patients with cancer [63,66,69,70,76,77], as well as individuals with colorectal [55], cardiovascular [56], and musculoskeletal [62] diseases. However, a pilot study [73] found that patients in the control group—who did not have access to the additional information provided by the mobile app—showed significantly lower anxiety and depression than the intervention population. As this particular study explored patients with cancer, it is possible that the increased amount of knowledge in the app reminded women of the cancer treatment they were going through. In contrast, a systematic review [38] reported that 17 studies found eHealth solutions to improve anxiety among patients with breast cancer. Nevertheless, other studies have reported eHealth interventions to exert contradictory effects on anxiety [40,83]. This includes the surprising finding that increased knowledge does not necessarily reduce anxiety. This area of research clearly needs to be investigated in more detail.

Various digital counseling approaches significantly improved adherence to treatment among patients requiring hemodialysis [78], as well as individuals with musculoskeletal [59], colorectal [55], and cardiovascular [74] diseases. In 23% (6/26) of the studies, although adherence to treatment increased among patients with cardiovascular [54,57,71] and colorectal diseases [60,61] and cancer [75], no statistically significant differences between the groups (intervention and control) were found. Digital interventions also significantly improved the clinical indicators related to adherence to treatment among patients with cardiovascular disease [58,68,72]. For example, eHealth interventions significantly improved adherence to treatment [84] and blood pressure control [29,37,41,42,45,85]. Recent systematic reviews of mHealth interventions for hypertension [28] and coronary artery disease [30] have provided evidence that mHealth interventions are effective for blood pressure control, self-management, and medication adherence. It should be noted that the overall risk of bias was relatively high in both these studies.

The lack of significant improvements in the outcomes of patients who are chronically ill after digital counseling interventions may be explained by various methodological issues such as a short follow-up period or insufficient power to detect changes in outcomes [31,33,34,85]. A recent umbrella review [31] concluded that telemedicine has the potential to improve clinical outcomes in patients with diabetes; however, it was not found to have a significant and clinically meaningful impact on blood pressure because the outcomes measuring blood pressure showed low overall certainty. Risk of bias; inconsistency; differences in patient populations, settings, and interventions; imprecision; publication bias; and the underreporting of relevant information have been listed as the main reasons why previous reports have only provided low-quality evidence concerning the effectiveness of digital counseling approaches. In addition, the heterogeneity in eHealth definitions also makes between-study comparisons difficult, which are necessary to provide health care professionals with evidence-based recommendations [30,48,86,87].

### Limitations

This review includes a few inherent limitations. The literature search conducted for this review excluded gray literature, which means that relevant studies may have been overlooked. Language limitations were not used during the search process, but only studies published in English, Finnish, and Swedish were considered during the screening process. This may have resulted in language bias.

A further limitation was the varying quality and heterogeneity of the selected studies, that is, sample sizes, type of interventions, and length of follow-up times, which differed among the studies. The sample sizes were small (fewer than 200 patients overall) in 65% (17/26) of the studies. The complex digital counseling interventions were diverse and heterogeneous in content and had various risks of bias in their methodology. Quality assessment was performed using a standardized Joanna Briggs Institute critical appraisal tool for RCTs and quasi-experimental studies [52] to avoid systematic bias. Of the 24 RCT studies, 9 (38%) scored at least 10 points out of 13, whereas 15 (62%) scored less than 10 points out of 13. Of the 24 RCT studies, 2 (8%) had the lowest score, 8 points out of 13. Both quasi-experimental studies were rated as good quality. The highest risk of bias in the selected studies related to blinding of participants and personnel, blinding of outcome assessment, and incomplete outcome data.

Of the 26 studies, 6 (23%) did not measure anxiety, depression, or adherence to treatment as a primary outcome of digital counseling interventions. In addition, several different scales were used to measure the selected primary outcomes. Because of the heterogeneity of the outcomes measured and scales used in the included studies, we could not perform a meta-analysis. This may have introduced additional bias in the results.

The review was strengthened by the use of a systematic and extensive search process that used several databases and was conducted with the assistance of an information specialist. To avoid subjective selection bias, studies were selected for inclusion by 2 researchers (KPP and MK) working independently.

### Conclusions

Among the 26 included studies, 10 (38%) digital, web-based interventions demonstrated statistically significant positive effects on anxiety and depression, adherence to treatment, and clinical indicators related to adherence to treatment. Positive, albeit statistically nonsignificant, changes were reported in 69% (18/26) of the studies. These results indicate that digital environments may improve anxiety, depression, and adherence to treatment among patients who are chronically ill, and hence have significant repercussions for the health care sector.

## Appendix

Multimedia Appendix 1 Search strategy for different databases.

Multimedia Appendix 2 Critical appraisal of the selected randomized controlled trial studies (N=24).

Multimedia Appendix 3 Details of the included studies.

## Abbreviations

HADS: Hospital Anxiety and Depression Scale
mHealth: mobile health
PRISMA: Preferred Reporting Items for Systematic Reviews and Meta-Analyses
RCT: randomized controlled trial
