# Supplementary material for: Effectiveness of Digital Counseling Environments on Anxiety, Depression, and Adherence to Treatment Among Patients Who Are Chronically Ill: Systematic Review
Source: J Med Internet Res. 2022 Jan 6;24(1):e30077. doi: 10.2196/30077 (PMC8778552; doi:10.2196/30077)
Supplement: Multimedia Appendix 1 [file jmir_v24i1e30077_app1.docx]

Multimedia Appendix 1. Search strategy for different databases

| Databases | Search strategy |
| --- | --- |
|  |  |
| **Ebsco Database**  **(CINAHL)** |  |
|  | (digital OR web* OR mobile OR virtual OR ubiquitous OR mhealth OR ehealth OR uhealth OR "virtual reality" OR "augmented reality" OR 360* OR online) AND (patient OR patients) AND (counsel* OR education OR guidance OR teaching OR information) AND (anxiety OR fear OR adherence to treatment OR adherenc*) AND (rct or randomized control trial or randomized controlled trial or randomised controlled trial or randomised control trial) OR quasi-expe*) |
|  |  |
| **PubMed** |  |
|  | ((((((digital OR web-based OR mobile OR virtual OR ubiquitous OR mhealth OR ehealth OR uhealth OR "virtual reality" OR "augmented reality" OR 360 OR online) AND (patient OR patients)) AND ((counsel*) OR education OR guidance OR teaching OR information)) AND (((rct OR "randomised control trial" OR "randomised controlled trial" OR "randomized controlled trial" OR quasi*)))) AND (((anxiety OR fear OR "adherence to treatment" OR adherenc*)))) |
|  |  |
| **Scopus** |  |
|  | ((digital OR web* OR mobile OR virtual OR ubiquitous OR mhealth OR ehealth OR uhealth OR "virtual reality" OR "augmented reality" OR 360* OR online) AND ((patient OR patients) W/15 (counsel* OR education OR guidance OR teaching OR information))) AND (rct OR "randomised control trial" OR "randomised controlled trial" OR "randomized controlled trial" OR quasi*) AND (anxiety OR fear OR "adherence to treatment" OR adherenc*) |
|  |  |
| **Web of Science** |  |
|  | (digital OR web* OR mobile OR virtual OR ubiquitous OR mhealth OR ehealth OR uhealth OR "virtual reality" OR "augmented reality" OR 360* OR online) AND (rct OR "randomised control trial" OR "randomised controlled trial" OR "randomized control trial" OR "randomized controlled trial" OR quasi*) AND ((patient or patients) NEAR (counsel* OR education OR guidance OR teaching OR information) AND (anxiety OR fear OR adherence to treatment OR adherenc*)) |
