# Supplementary material for: Effectiveness of Digital Counseling Environments on Anxiety, Depression, and Adherence to Treatment Among Patients Who Are Chronically Ill: Systematic Review
Source: J Med Internet Res. 2022 Jan 6;24(1):e30077. doi: 10.2196/30077 (PMC8778552; doi:10.2196/30077)
Supplement: Multimedia Appendix 2 [file jmir_v24i1e30077_app2.docx]

| Study | Criteria | | | | | | | | | | | | | Score out of 13 |
| --- | --- | --- | --- | --- | --- | --- | --- | --- | --- | --- | --- | --- | --- | --- |
|  | Randomization^a^ | Concealed allocation from groups^b^ | Similarity in treatment groups^c^ | Blinding of participants^d^ | Blinding of those delivering treatment^e^ | Blinding of outcomes assessors^f^ | Identical treatment of groups^g^ | Complete follow-up^h^ | Intention-to-treat analysis^i^ | Similarity in outcome measurements^j^ | Reliability of outcome measurements^k^ | Appropriate statistical analysis^l^ | Appropriate trial design^m^ |  |
|  | | | | | | | | | | | | | | |
| Børøsund et al [53] | Yes | Yes | Yes | No | Yes | Yes | Yes | Yes | Yes | Yes | Yes | Yes | Yes | 12 |
| Choi et al [54] | Yes | Uc^n^ | Yes | Uc | Uc | Uc | Yes | Yes | Yes | Yes | Yes | Yes | Yes | 9 |
| Elkjaer et al [55] | Yes | Yes | Yes | Yes | Yes | No | Yes | Yes | Yes | Yes | Yes | Yes | Yes | 12 |
| Foley et al [73] | Yes | Uc | Yes | Uc | Uc | Uc | Yes | Yes | No | Yes | Yes | Yes | Yes | 9 |
| Greer et al [69] | Yes | No | Yes | No | Uc | Yes | Yes | Yes | Uc | Yes | Yes | Yes | Yes | 9 |
| Guo et al [74] | Yes | No | Yes | No | No | Yes | Yes | Yes | Uc | Yes | Yes | Yes | Yes | 9 |
| Habibovic et al [56] | Yes | Yes | Yes | No | No | Yes | Yes | Yes | Yes | Yes | Yes | Yes | Yes | 11 |
| Handa et al [70] | Yes | No | Yes | No | No | No | Yes | Yes | Yes | Yes | Yes | Yes | Yes | 9 |
| Helzlsouer et al [75] | Yes | Yes | Yes | No | No | Uc | Yes | Yes | Uc | Yes | Yes | Yes | Yes | 9 |
| Keyserling et al [57] | Yes | Uc | Yes | Uc | Uc | Uc | Yes | Yes | Uc | Yes | Yes | Yes | Yes | 8 |
| Kim et al [58] | Yes | No | Yes | Yes | No | Uc | Yes | Yes | Uc | Yes | Yes | Yes | Yes | 9 |
| Lambert et al [59] | Yes | Yes | Yes | No | No | Yes | Yes | Yes | Yes | Yes | Yes | Yes | Yes | 11 |
| Liu et al [68] | Yes | No | Yes | No | No | Yes | Yes | N/A^o^ | No | Yes | Yes | Yes | Yes | 8 |
| Liu et al [72] | Yes | No | Yes | Yes | Yes | Uc | Yes | Yes | Yes | Yes | Yes | Yes | Yes | 11 |
| Mata et al [60] | Yes | Yes | Yes | No | Yes | Yes | Yes | Yes | Yes | Yes | Yes | Yes | Yes | 12 |
| Persell et al [67] | Yes | Yes | Yes | No | No | No | Yes | Yes | Yes | Yes | Yes | Yes | Yes | 10 |
| Petzel et al [76] | Yes | Yes | Yes | Yes | Yes | Uc | Yes | Uc | No | Yes | Yes | Yes | Yes | 10 |
| Sharara et al [61] | Yes | Uc | Yes | No | Uc | Yes | Yes | Yes | Uc | Yes | Yes | Yes | Yes | 9 |
| Strøm et al [62] | Yes | Yes | Uc | No | No | No | Yes | Yes | No | Yes | Yes | Yes | Yes | 9 |
| Urech et al [66] | Yes | Uc | Yes | No | No | Uc | Yes | Yes | Yes | Yes | Yes | Yes | Yes | 10 |
| White et al [63] | Yes | Uc | Yes | No | Uc | Uc | Yes | Yes | Yes | Yes | Yes | Yes | Yes | 9 |
| Widmer et al [64] | Yes | Uc | Yes | No | No | Yes | Yes | Yes | Uc | Yes | Yes | Yes | Yes | 9 |
| Yun et al [65] | Yes | No | Yes | Uc | Uc | Uc | Yes | Yes | Yes | Yes | Yes | Yes | Yes | 9 |
| Yu et al [71] | Yes | No | Yes | No | No | No | Yes | Yes | Yes | Yes | Yes | Yes | Yes | 9 |

^a^Was true randomization used for assignment of participants to treatment
groups?

^b^Was allocation to treatment groups concealed?

^c^Were treatment groups similar at the baseline?

^d^Were participants blind to treatment assignment?

^e^Were those delivering treatment blind to treatment assignment?

^f^Were outcomes assessors blind to treatment assignment?

^g^Were treatment groups treated identically other than the intervention of
interest?

^h^Was follow-up complete, and if not, were differences between groups in
terms of their follow-up adequately described and analyzed?

^i^Were participants analyzed in the groups to which they were randomized?

^j^Were outcomes measured in the same way for treatment groups?

^k^Were outcomes measured in a reliable way?

^l^Was appropriate statistical analysis used?

^m^Was the trial design appropriate, and were any deviations from the standard randomized controlled trial design (individual randomization and parallel groups) accounted for in the conduct and analysis of the trial?

^n^Uc: unclear.

^o^N/A: not applicable.
