# Supplementary material for: Effectiveness of Digital Counseling Environments on Anxiety, Depression, and Adherence to Treatment Among Patients Who Are Chronically Ill: Systematic Review
Source: J Med Internet Res. 2022 Jan 6;24(1):e30077. doi: 10.2196/30077 (PMC8778552; doi:10.2196/30077)
Supplement: Multimedia Appendix 3 [file jmir_v24i1e30077_app3.docx]

| ­Author (year), country | Aim | Participants | Design | Intervention | Main findings | Outcomes | Quality asses-ment score |
| --- | --- | --- | --- | --- | --- | --- | --- |
| Børøsund et al.  (2014) Norway  [53] | to compare the effects of (1) an Internet-based patient provider communication service (IPPC), (2) WebChoice, a Web-based illness management system for breast cancer patients (IPPC included), and (3) usual care on symptom distress, anxiety, depression | patients recently diagnosed with breast cancer and undergoing treatment  (n=167) | rct,  multi-center study  duration: 12 months | WebChoice group (n=64): symptom monitoring, tailored information and self-management support, a diary, and communication with other patients  The IPPC group (n=45): nurse-administered IPPC: sending secure e-messages to and receive e-messages from health care personnel  control: usual care group | WebChoice group reported significantly lower anxiety (mean difference 0.79, 95% CI 0.09-1.49, *P*=.03), and depression (mean difference 0.79, 95% CI0.09-1.49, *P*=.03) compared with the usual care group.  The IPPC group reported significantly lower depression scores compared with the usual care group (mean difference 0.69, 95% CI 0.05-1.32, *P*=.03), no differences observed for symptom distress or anxiety. | primary: symptom distress, anxiety, depression  secondary: self-efficacy | 12/  13 |
| Choi et al  (2019)  USA  [54] | to test if asynchronous dietary counseling supplied through a custom smartphone app results in better adherence to a Mediterranean diet in a non-Mediterranean population than traditional counseling | cardiology patients with ongoing cardiology care with a cardiologist that is expected to continue for at least 6 months  (n=100) | pilot rct  duration: 6 months | smartphone app-based experimental (IG) Mediterranean diet intervention: meal diary, exercise tracking, BP monitoring  control (CG):  2 additional sessions of in-person dietary counseling with the registered dietitian—30 min at 1 month and 30 min at 3 months | no significant differences between groups with BP, lipid parameters, hemoglobin A1c, or C-reactive protein (CRP). Participants in IG achieved a significantly greater weight loss on average of 3.3 pounds versus 3.1 pounds for participants in CG, *P*=.04. Adherence to the Mediterranean diet increased significantly over time for both groups (*P*<.001), no significant difference between groups (*P*=.69). The proportion of participants with high Mediterranean diet compliance (defined as the MDS ≥9) increased significantly over time (*P*<.001)—from 18.4% to 57.1% for CG and 27.5%to 64.7% for IG; however, there was no significant difference between the groups. | primary: adherence to the Mediterranean diet  secondary: BP, weight, fasting lipid parameters (total cholesterol, HDL cholesterol, LDL cholesterol, and triglycerides), HbA1c, high-sensitivity CRP, participant satisfaction with the intervention, and percent of participants achieving high compliance with a Mediterranean diet (defined as MDS ≥9). | 9/  13 |
| Elkjaer et al  (2010)  Denmark, Ireland  [55] | to 1.) investigate whether a self-administered, web-based e-health treatment programme was feasible for patients with ulcerative colitis on 5-ASA therapy, 2.) to determine whether this e-health approach could improve patient compliance, knowledge, QoL, disease outcomes, and safety and 3.) to investigate whether the e-health approach could decrease healthcare costs primarily in Denmark | patients with mild/moderate ulcerative colitis and 5-aminosalicylate acid treatment  (n=333) | rct,  two center  study  duration: 12 months | Web platform (IG):  contact to the web-doctor by secure mail or call/send a text message to the web-doctor’s cell phone. Patient status indicator, disease activity and QoL graphs  control (CG): conventional follow-up in the IBD out-patient clinic: routine appointments, and the possibility to advance appointments | Adherence to 4 weeks of acute treatment was increased by 31% in Denmark and 44% in Ireland compared to the CG. In Denmark IBD knowledge and QoL were significantly improved in IG. Median relapse duration was 18 days (95% CI10 to 21) in the IG versus 77 days (95% CI 46 to 108) in the CG. | patients’ compliance, knowledge, quality of life (QoL), anxiety, disease outcomes, safety and health care costs | 12/  13 |
| Fang et al  (2019)  Taiwan  [77] | to evaluate the effect of a web-based survivorship care plan computerized application for women with breast cancer | Women diagnosed with breast cancer, who had completed their primary treatment but less than 5 years without a sign of recurrence (n=165) | quasi-experimental study  duration: 12 months | mobile application (IG): personalized information presented in 7 modules using texts or videos, push notifications reminding to take prescribed medicine, participate in a module, seek remedies for side effects, and the date of next out patient follow-up.  control (CG): educational leaflet | a significant decrease in women in the IG group compared to the CG for total unmet needs since T3 (*P*<.004) and fear of recurrence since T4 (*P*=.02). Women in the IG group also reported significant improvements in QoL at T4 (*P*<.001) relative to those in the CG. | primary: unmet needs  secondary: fear of recurrence, symptom distress, anxiety, depression, and quality of life (QoL) | 8/9 |
| Foley et al.  (2016)  Ireland  [73] | to evaluate the effects of a mobile information application on  anxiety levels of patients undergoing surgery for breast cancer | adult women undergoing surgical  procedures for breast cancer (n=39) | pilot rct,  1:2 allocation  duration: 2 weeks | mobile application (IG):  Individually tailored disease information, multimedia graphics, animations  control (CG): standard information | Anxiety and depression scores at seven days significantly lower in CG without access to the additional information provided by the mobile application | primary: anxiety levels and depressive symptoms  secondary: coping styles and strategies in patients with  cancer | 9/13 |
| Greer et al.  (2019)  USA  [69] | to test the efficacy of a tailored cognitive-behavioral therapy mobile application to treat anxiety in patients with incurable cancer | Adult patients with incurable cancers and elevated anxiety symptom  (n=145) | rct,  two center study  duration: 3 months | Mobile application with tailored programming logic, videos, homework exercises  Control: a health education program administered over a tablet during the12-week study period, no tailored programming logic, videos, or homework assignments. | Both the IG for anxiety and the CG were associated with improvements in anxiety, mood, and QOL, but these outcomes did not differ between study groups. The IG was more beneficial than CG for patients with severe baseline anxiety. | primary: anxiety, depression symptoms, and quality of life (QOL)  secondary: Clinical Global Impression Scale, Patient Health, and Functional Assessment of Cancer Therapy | 9/13 |
| Guo et al. (2017)  China  [74] | to perform a randomized, controlled trial  of a Mobile Health technology–supported atrial  fibrillation management model, integrating clinical decision  support tools, guideline-based treatment, and patient involvement | adult patients with atrial fibrillation (n=113) | pilot rct,  two center study  duration: 3 months | mobile application (IG):  educational program (8 components): disease information, with additional patient self-support items), personal health record with individual recommendations, patient involvement self-care components, and structured follow-up components, alerts  control (CG): usual care | Significant improvements in knowledge compared with the CG (*P*-values for trend<.05). Drug adherence and anticoagulant satisfaction significantly better with the IG versus CG (all *P*<.05). Quality of life scores significantly increased in the IG versus CG, with anxiety and depression reduced (all *P*<.05). | primary: patients’ knowledge  secondary: quality of life, drug adherence, and anticoagulation satisfaction | 9/13 |
| Habibovic  (2017)  Netherlands  [56] | to evaluate the long-term effects of WEBCARE, focusing on (a) psychological disease-specific and generic outcome measures, and (b) which patients benefit the most based on subgroup analyses | patients with implantable cardioverter defibrillator (ICD) from 6 hospitals  (n=289) | rct  duration: 12 months | Webcare (IG): an online, 12-weeks fixed, 6 lesson behavioral treatment based on problem solving therapy: information, six preprogrammed lessons, feedback, CD with relaxation exercises  control (CG): care as usual | No significant difference on anxiety, depression or quality of life between the IG and CG at 6- and 12-months post implantation. | anxiety, depression, quality of life, personality, ICD related concerns, shock anxiety, device acceptance | 11/  13 |
| Handa et al  (2020)  Japan  [70] | to test the effectiveness of a smartphone application (app) as a support tool | Patients undergoing breast cancer chemotherapy  (n=102) | rct  duration: 12 weeks | smartphone application: Adverse events reporting, tips and recommendations on self-care  control: written ordinary materials | No significant improvement was seen in anxiety, depression, or health literacy at the end of treatment between the two groups | primary: anxiety and depression  secondary: health literacy, side effects, app adherence | 9/13 |
| Helzlsouer et al  (2016)  USA  [75] | to assess the feasibility of an interdisciplinary centralized virtual navigation program to support treatment completion among newly diagnosed low-income patients with breast cancer | newly diagnosed, stage 0 to III breast cancer patients whose treatment plans included adjuvant treatment beyond surgery  (n=101) | pilot rct  duration: 12 months | web-based application (IG) with individually tailored information and navigation program with nurse/social worker support by phone  control (CG): web-based information access | More patients on the IG completed treatment compared with those in the CG, but this difference was not statistically significant. | Adherence to recommended chemotherapy, radiation therapy, and/or initiation of hormone therapy unscheduled outpatient visits, emergency department (ED) visits, and hospitalization  pain, mood, distress, and fatigue | 9/13 |
| Keyserling et al  (2014)  USA  [57] | to assess the effectiveness, acceptability, and cost-effectiveness of a combined L&M intervention to reduce CHD risk offered in counselor-delivered and web-based formats | patients at moderate to high risk for Coronary Heart Disease (10-year Framingham Risk Score [FRS] ≥10%)  (n=385) | rct  duration: 12 months | Website (IG):  web-based decision aid plus web-based counseling program,  pedometer for self-monitoring  control (CG): web-based decision aid plus counseling program by counselor, pedometer for self-monitoring | In both intervention arms CHD risk reduced through the 12-month follow-up period, yet no significant between-group differences were found. | primary: change in FRS at 4-month follow-up  secondary: blood pressure, blood lipid levels, lifestyle behaviors, and medication adherence, Acceptability and cost-effectiveness | 8/13 |
| Kim et al. (2016)  USA  [58] | to study the influence of wireless self-monitoring program and patient activation measures on health behaviors,  medication adherence, and blood pressure levels of hypertensive patients. | hypertensive patients (n=95) | rct  duration: 6 months | Combination (IG):  HealthyCircles Platform: an online disease management program featuring educational materials, consumer portals, and a dashboard to link.  A mobile app: reminders for self-monitoring, monitoring device connected with a mobile phone, importing health data  control (CG): standard disease management program | Improvements in patient activation were associated with improvements in cigarette smoking and blood pressure control. This relationship was further strengthened in reducing cigarettes,  alcohol drinking, and systolic and diastolic blood pressure at 6 months among individuals participating in the wireless self-monitoring program. | primary: health behaviors (frequency of the use of alcohol, smoking, and exercise)  secondary: medication adherence, blood pressure | 9/13 |
| Lambert  (2017)  Australia  [59] | to investigate the potential of an app to promote adherence to HEPs in an effort to optimise patient outcomes | patients with an upper or lower limb injury or condition, had been provided with 4 weeks of home exercises by a physiotherapist  (n=80) | rct  duration: 4 weeks | Mobile application (IG) with home exercise programs, supplementary phone calls and motivational text messages  control (CG): home exercise programs as a paper handout | participants in the IG reported greater adherence and greater improvements in function compared to CG. The mean between-group difference for self-reported exercise adherence was 1.3 (11 points; 95% CI 0.2-2.3) in favor of the IG, which was a statistically significant result (*P*=.01). | primary: self-reported exercise adherence  secondary: captured functional performance, disability, patient satisfaction, perceptions of treatment effectiveness, and different aspects of adherence | 11/  13 |
| Liu et al.  (2019)  China  [68] | to explore the short-term effectiveness of a self-empowerment app in supporting people with CVD on lipid control and medication adherence | Patients with cardiovascular disease  (n=57) | rct  duration: 3 months | Mobile application (IG) with health education (tailored health plan and daily updates on lifestyle, diet, and treatment), medication reminders, medication recording and daily feedback, self-empowerment via automatic real-time video feedback based on the subjects’ adherence, weekly text messages on health education  control (CG): weekly text messages on health education | In the IG significant reductions in both triglyceride and total cholesterol levels relative to the CG (*P*=.020 and .014), Medication adherence also significantly increased in the IG (43.33% vs 82.14%, *P*=.002), compared to the CG (30.00% vs 37.93%, *P*=.520). This between-group difference was statistically significant (82.14% vs 37.93%, *P*=.001). | primary: low-density lipoprotein cholesterol (LDL), total cholesterol (TC), triglyceride (TG), and high-density lipoprotein cholesterol (HDL).  secondary: medication adherence, and the occurrence of major adverse cardiac events (MACE) | 8/13 |
| Liu et al (2020)  Canada  [72] | to evaluate the efficacy of e-Counseling to promote lifestyle behaviors, 2) examine whether these behaviors changes were associated with lower blood pressure (BP), and Framingham Risk Index | Hypertensive  patients  (n=264) | rct, multi-center  study  duration: 12 months | webpage (IG): videos, online handouts, self-monitoring tools and self-help resources, self-help plan, feedback, virtual peer support  control (CG): 28 email newsletter articles | e-counseling resulted in a significant reduction in systolic blood pressure after 12 months when compared to the CG (-10.1mmHg [95% (CI), -12.5,-7.6] versus -6.0mmHg [95%CI,-8.5,-3.5]; *P*=.02) | primary: BP  secondary: exercise and diet behaviors | 11/  13 |
| Mata et al  (2019)  Canada  [60] | to estimate the extent to which a novel mobile device app affects adherence to an ERP for colorectal surgery in comparison to standard written education. | patients with colonic or rectal diseases undergoing colorectal resection  (n=97) | rct  duration: 3 months | iPad including a novel mobile device app (IG) for postoperative education and self-assessment of recovery: checklist,  Daily clinical questionnaires, education modules  plus standard preoperative education  control (CG): standard preoperative education (education session with nursing personnel and an illustrated booklet) and iPad without the app | access to a mobile health application did not improve adherence to a well-established enhanced recovery pathway in colorectal surgery patients, when compared to standard written patient education. | primary: mean adherence (%) to a bundle of five postoperative ERP elements: mobilization, gastrointestinal motility stimulation, breathing exercises, and consumption of oral liquids and nutritional drinks.  secondary: Length of primary hospital stay, Intraoperative and postoperative complications, emergency department visits, Patient satisfaction | 12/  13 |
| Park et al  (2019)  Korea  [78] | to evaluate the effects of an integrated self-management program using a mobile application among hemodialysis patients | hemodialysis patients  (n=84) | A quasi-experimental study  duration: 8 weeks | mobile application (IG), SMS messages twice per week, and face-to-face counseling and education twice per month, interactive bulletin board that was linked with the mobile application and SMS  control (CG): usual care | The use of a mobile application significantly improved self-efficacy when compared to the results from the CG (4.79±3.51 vs. −1.05±2.05; t=−9.30, *P*<.001). Treatment compliance also significantly increased in the IG (11.57±7.63) relative to the CG (−1.74±2.71; t=−10.66, *P*=.001). | Self-efficacy, treatment compliance, ratio of interdialytic weight gain to dry weight | 8/9 |
| Persell et al  (2020)  USA  [67] | to investigate the effect of an artificial intelligence smartphone coaching app to promote home monitoring and hypertension-related behaviors on systolic blood pressure level | Participants with uncontrolled hypertension  (n= 333) | rct  duration: 6 months | a smartphone coaching app (IG) to promote home monitoring and behavioral changes associated with hypertension self-management plus a home blood pressure monitor  Control (CG): a blood pressure tracking app plus a home blood pressure monitor | Among individuals with uncontrolled hypertension, those randomized to a IG had similar systolic blood pressure  compared with those in CG. | primary: systolic blood pressure  secondary: antihypertensive medication adherence, home monitoring and self-management practices, self-efficacy associated with blood pressure, weight, and self-reported health behaviors. | 10/  13 |
| Petzel et al  (2018)  USA  [76] | to evaluate the effectiveness of a patient-centered information-based website, designed to promote cancer education, emotional well-being and self-management for women with advanced ovarian cancer | women with advanced ovarian cancer (stages III-IV), scheduled for a post-operative checkup or planned chemotherapy visit  (n=35) | pilot rct  duration: 3 months | website Together (IG): a learning library with tailored information, distress self-monitoring, medical information  control (CG): usual care materials delivered as PDF files via a separate website, paper materials | no differences between the IG and CG were observed for any outcomes, although the IG demonstrated lower, but non-significant, general distress. | psychological distress, anxiety, depression, and knowledge about ovarian cancer | 10/  13 |
| Sharara et al. (2017)  Lebanon  [61] | to assess the effect of a customized mobile application on adherence with instructions and quality of graded bowel preparation | adult patients (aged over 18) scheduled for elective colonoscopy (n=160) | rct  duration: 3 days | mobile application (IG): instructions, examples and photographs, daily push notifications, verification feature  + paper instructions  control (CG): paper instructions | Complete adherence with instructions reported in 73.4% of CG vs. 82.4% of IG (*P*=0.40).  No significant difference observed in patient overall compliance and bowel cleanliness between both arms as measured by the three bowel preparation scales.  The App was user-friendly and received higher overall rating in this respect than paper instructions. | primary: adherence with instructions.  secondary: Quality of preparation. | 9/13 |
| Strøm et al  (2019)  Denmark  [62] | to examine the effect of a web-based Spine Platform featuring Interaction and Information by Animation on symptoms of anxiety and depression, pain, disability, and health-related quality of life. | patients scheduled for instrumented lumbar spine fusion due to degenerative disc disease or spondylolisthesis  (n=114) | rct  duration: 3 months | web-based Spine Platform (IG): animated information, interaction, an internet support group (ISG) and a diary  plus standard information  control (CG): standard information, 2-hour joint session 1 to 5 week before surgery | There was no statistically significant difference within the IG and the CG regarding changes in HADS at 3-month follow-up (*P*≥.37). | primary: the change in self-reported Hospital Anxiety and Depression Scale  secondary: change in HADS 1-day before surgery, self-reported disability, quality of life, and the low back pain | 9/13 |
| Urech et al  (2018)  Switzerland  [66] | to assess feasibility and efficacy of Web-based stress management for newly diagnosed patients with cancer | patients with cancer who had started first-line treatment within the previous 12 weeks  (n=129) | rct  duration: 2 months | web-based intervention (IG): eight modules with stress reduction techniques, downloadable audio files with relaxation and guided-imagery exercise, feedback  control (CG): cancer treatment locally as planned | quality of life was significantly higher (mean, 8.59 points; 95% CI, 2.45 to 14.73 points; *P*= .007) and distress significantly lower (mean,20.85; 95% CI,21.60 to20.10; *P*=.03) in the IG as compared with the CG. Changes in anxiety or depression were not significant (mean,21.28;95% CI,23.02 to 0.45; *P*= .15). | primary: quality of life after the intervention  secondary: distress and anxiety or depression | 10/13 |
| White et al  (2018)  Australia  [63] | to assess the effectiveness of a purpose- built information- based website to reduce distress among young women with breast cancer | young, female breast cancer survivors approximately 6 months post-diagnosis  (n=337) | rct  duration: 6 months | The “informe” website (IG): information, distress thermometer, feedback, diary  control (CG): usual care from treating clinicians | The mean levels of anxiety or depression did not significantly differ between the IG and CG. | primary: anxiety and depression  secondary: quality of life (QoL), unmet information needs | 9/13 |
| Widmer et al. (2017)  USA  [64] | to determine whether digital health intervention during cardiac rehabilitation (DHI) administered during cardiac rehabilitation (CR) would reduce CV-related emergency department (ED) visits and rehospitalizations in patients after percutaneous coronary intervention (PCI) for acute coronary syndrome (ACS) | patients undergoing cardiac rehabilitation following acute coronary syndrome and percutaneous coronary intervention  (n=80) | rct  duration: 12 weeks | Combination (IG):  educational information, reporting of health behavior and importing health data, personalized feedback  control (CG): standard rehabilitation | The IG had improved weight loss compared to the CG. Those in the IG also showed a non-significant reduction in CV-related rehospitalizations plus ED visits compared to the CG at 180 days. | primary:  CV-related emergency department (ED) visits and re-hospitalizations,  secondary: blood pressure, height, weight, health behavior | 9/13 |
| Yun et al  (2012)  Korea  [65] | to determine whether an Internet-based tailored education program is effective for disease-free cancer survivors with cancer-related fatigue | patients who had completed primary cancer (stages I -III) treatment within the past 24 months, had reported moderate to severe fatigue for at least 1 week  (n=273) | rct  duration: 12 weeks | Website (IG): individually tailored education program: self-assessment and graphic reports, health advice and online education, enhanced and short message services, caregiver monitoring and support, and health professional monitoring.  control (CG): | The intervention significantly decreased patients’ Anxiety and Depression score (-0.90; 95% CI,-1.51 to-0.29) relative to the CG.  The IG had a significantly greater decrease in fatigue compared to CG. | primary: Fatigue  secondary: Anxiety and Depression, Quality of Life | 9/13 |
| Yu et al  (2020)  China  [71] | to evaluate the effectiveness and feasibility of using a smartphone-based application to improve medication adherence in patients after coronary artery bypass grafting | patients who underwent isolated coronary artery bypass grafting  (n= 1000) | rct, multi-center  study  duration: 6 months | the smartphone application (IG): educational material, automatic reminders, goal setting, feedback, encouragement, and advice, individual risk factor control levels  control (CG): standard post-CABG care: cardiology education, instruction on secondary prevention, and promotion of self-care management during the inpatient stay | The study did not reveal any significant between-group differences in mean medication adherence scores (mean difference 0.052, 95CI-0.087 to 0.191, *P*=.460) at the 6-month follow-up point. | primary: preventive medication adherence  secondary: mortality, major adverse cardiovascular and cerebrovascular events, cardiovascular rehospitalization, self-reported secondary preventive medication use, blood pressure, body mass index, smoking status | 9/13 |

a Abbreviations:

IG=Intervention group, CG=Control group
